# Personals

**Published:** 1919-01

**Authors:** 


### Personals.

Dr. Edgerton, of Rio Grande City has announced that he will open an office in McAllen in the very near future.

---

Dr. John W. Black, of Hearne, Texas, has just returned from New Orleans, where he has been doing post-graduate work in Tulane University. Much to the regret of his many friends, he is leaving Hearne and moving to Bryan, where he has accepted a partnership with Dr. W. H. Oliver, and will become connected with the Bryan Sanitarium.

---

Dr. T. E. Tidd, formerly located at Crystal City, has moved to

Staples. Dr. Tidd will look after the practice of Dr. B B. Liles, who went into military service several months ago.

---

Dr. R. L. Howell, of Snyder, Texas, who has just returned war service has moved to Brownwood, where he will engage in the practice of medicine.

---

Gertrude Lustig, former head nurse at the Camp Bowie Base Hospital, is now confined in the enemy alien quarters on Ellis Island, near New York City. The former nurse was taken from Dallas in company with several other enemy aliens about two weeks ago in charge of a United States Deputy Marshal and will remain on Ellis Island until her case is finally decided by the Federal authorities.

---

Dr. Collins, of Weston, is now located at Celina, Texas.

---

Dr. Otto Potthast, who was a lieutenant in the medical branch of the army awhile, has received his discharge and expects to from war service has moved to Brownwood, where he will engage in the practice of medicine.

---

Dr. James F. Duncan, formerly of Houston, Texas, is now located at Burkburnett where he is looking after his oil interests.

---

After an illness of two weeks, first assistant City Health Officer, Dr. V. P. Armstrong, of Dallas, has sufficiently recovered to resume his duties as head of the Emergency Hospital staff.

---

Dr. T. B. Taylor, of Elgin, has moved to Bastrop, Texas.

